# The Voltage Dependent Sidedness of the Reprotonation of the Retinal Schiff Base Determines the Unique Inward Pumping of Xenorhodopsin

**DOI:** 10.1002/anie.202103882

**Published:** 2021-09-15

**Authors:** Juliane Weissbecker, Chokri Boumrifak, Maximilian Breyer, Tristan Wießalla, Vitaly Shevchenko, Thomas Mager, Chavdar Slavov, Alexey Alekseev, Kirill Kovalev, Valentin Gordeliy, Ernst Bamberg, Josef Wachtveitl

**Affiliations:** ^1^ Department of Biophysical Chemistry Max-Planck-Institute of Biophysics Max-von-Laue-Straße 3 60438 Frankfurt am Main Germany; ^2^ Institute of Physical and Theoretical Chemistry Goethe University Max-von-Laue-Straße 7 60438 Frankfurt am Main Germany; ^3^ Institute of Biological Information Processing (IBI-7: Structural Biochemistry) Forschungszentrum Jülich GmbH Wilhelm-Johnen-Straße 52425 Jülich Germany; ^4^ European Molecular Biology Laboratory Notkestraße 85 22607 Hamburg Germany; ^5^ Research Center for Molecular Mechanisms of Aging and Age-related Diseases Moscow Institute of Physics and Technology Dolgoprudny Russia

**Keywords:** accessibility switch, inward proton pump, microbial rhodopsin, optogenetics

## Abstract

The new class of microbial rhodopsins, called xenorhodopsins (XeRs),^[1]^ extends the versatility of this family by inward H^+^ pumps.^[2–4]^ These pumps are an alternative optogenetic tool to the light‐gated ion channels (e.g. ChR1,2), because the activation of electrically excitable cells by XeRs is independent from the surrounding physiological conditions. In this work we functionally and spectroscopically characterized XeR from Nanosalina (*Ns*XeR).^[1]^ The photodynamic behavior of *Ns*XeR was investigated on the ps to s time scale elucidating the formation of the J and K and a previously unknown long‐lived intermediate. The pH dependent kinetics reveal that alkalization of the surrounding medium accelerates the photocycle and the pump turnover. In patch‐clamp experiments the blue‐light illumination of *Ns*XeR in the M state shows a potential‐dependent vectoriality of the photocurrent transients, suggesting a variable accessibility of reprotonation of the retinal Schiff base. Insights on the kinetically independent switching mechanism could furthermore be obtained by mutational studies on the putative intracellular H^+^ acceptor D220.

## Graphical abstract

The unique behaviour of inward proton pumping xenorhodopsins (XeR) makes these microbial rhodopsins an alternative optogenetic tool to the passively transporting light‐gated ion channels (e.g. channelrhodopsin‐1 and channelrhodopsin‐2), because the activation of electrically excitable cells by XeRs is independent from the surrounding physiological conditions. Here, we functionally and spectroscopically characterized XeR from Nanosalina (*Ns*XeR).

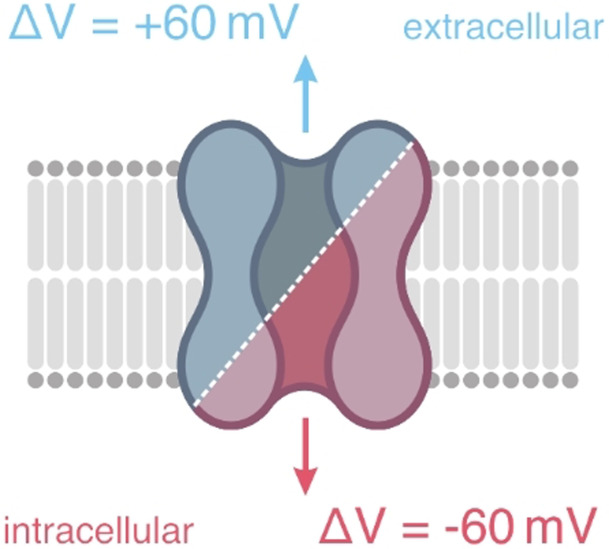

## Introduction

The task of photoreceptors for example, microbial rhodopsins (MRs) is to utilize light for energy conversion and sensory transduction.[^5^, ^6^] They share a 7‐transmembrane helix motif with a retinal chromophore bound via the Schiff base (SB) to the 7th helix.[^5^, ^6^] However, MRs are functionally diverse and come as pumps, channels, enzymes and light‐sensors. The protonation and deprotonation of the SB is typically a key process for their function. For ion pumps it regulates their vectoriality.

**Figure 1:**
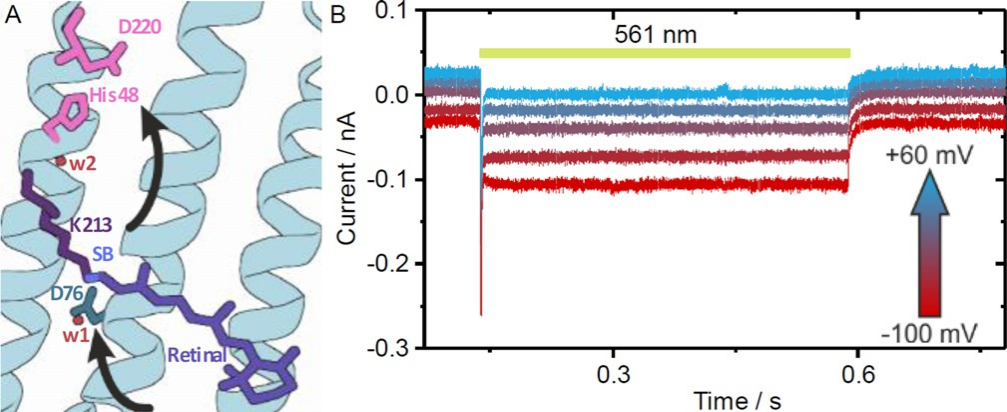
A) Binding pocket of *Ns*XeR with its chromophore, H^+^ acceptor pair D220/H48, H^+^ donor D76 and the water molecules w1 and w2 (arrows indicate the putative pathway of inward pumping). B) Light‐induced inward H+ pump currents by *Ns*XeR expressed in NG108‐15 cells (illumination period indicated by yellow bar).

In optogenetic applications light‐gated channels and light‐driven ion pumps are the fundamental tools to control neurons and muscle cells with high spatiotemporal resolution.^[7]^ The excitation of neurons is accomplished by use of channelrhodopsins (ChRs) that depolarize the cells upon illumination.[^8^, ^9^] Inhibition of the cells is achieved by hyperpolarization via light‐driven ion pumps. As an alternative a combination of inward and outward proton pumps could be used. These are independent from the cation gradient^[2]^ and could be used for hyperpolarization an depolarization as well. In the past, only outward proton pumps like bacteriorhodopsin bR^[10]^ and proteorhodopsin PR^[11]^ were known. However, a new class of MRs called xenorhodopsins (XeRs) was discovered recently,^[1]^ whose members are natural inward H^+^ pumps.[^2^, ^3^, ^4^] In XeRs, a putative H^+^ acceptor pair H48/D220 and the donor D76 (Figure 1 A) were identified by structural analysis,^[2]^ but the underlying mechanism of the pumping process is still unclear. *Ns*XeR as a minimally invasive optogenetic tool is appealing, because its dependence on the electrochemical gradient is negligible compared to that of the passive transporting ChRs. Therefore, in order to obtain deeper understanding of the molecular mechanism a thorough spectroscopic and electrophysiological study was performed in the present work. Specifically, the unusual quenching by blue light allowed insights into the molecular properties of the switch, which represents the key function for the vectoriality of H^+^ pumping.

## Results

**Figure 2:**
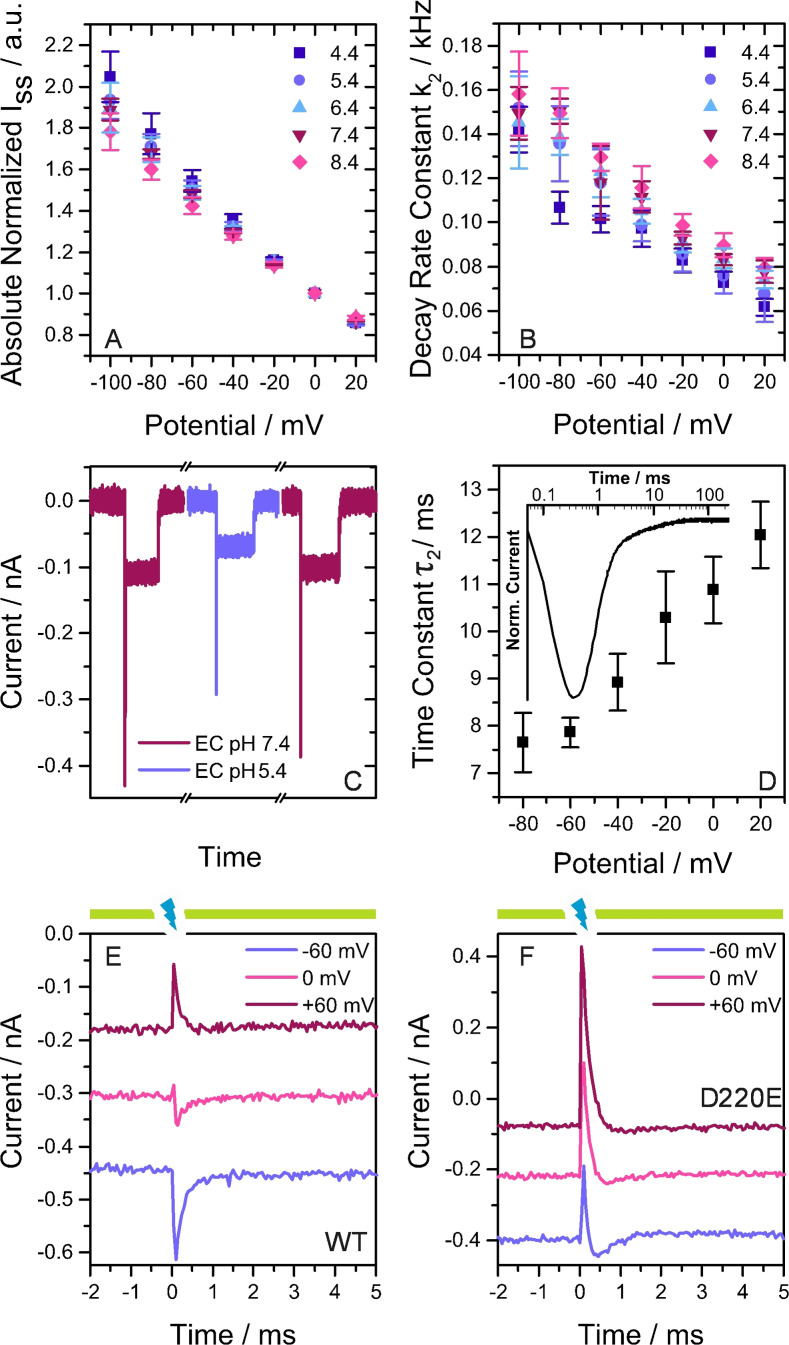
A) Absolute stationary photocurrents at different IC pH and EC pH 7.4 normalized to the currents at 0 mV. B) Exponential decay rate constants at differing IC pH and EC pH 7.4. A,B) Results are given as mean ± standard deviation (SD) with: *n*=4 (4.4), 4 (5.4), 4 (6.4), 3 (7.4) and 5 (8.4). C) Photocurrents at IC pH 7.4 and EC solution exchange measured on the same cell. D) Voltage dependence of the exponential time constant *τ*_2_ from ns illumination experiments on the WT at IC and EC pH 7.4 (*n*=5). The inset shows the normalized photocurrent transient of *Ns*XeR WT at 0 mV. E,F) Continuous 561 nm illumination (yellow bars) causes inward photocurrents and laser flashes (7 ns) at 355 nm (blue flash) cause transients.

The photocurrents of *Ns*XeR were investigated at different membrane potentials and pH values (Figure 1 B). For whole‐cell patch‐clamp experiments, *Ns*XeR was expressed in NG108‐15 cells and activated with yellow light (*λ*=561 nm). The resulting inward photocurrents (Figure 1 B) at different membrane potentials show a fast peak current that decays to a stable stationary photocurrent which relaxes back to the dark current level when the light is turned off. The normalized stationary photocurrents (*I*_ss_) showed a linear voltage dependence (Figure 2 A) that can be extrapolated to an apparent reversal potential of 118±6 mV (pH 7.4, *n*=3). The *I*_ss_ was not influenced by the pH of the intracellular (IC) solution. For the kinetic information, the decay of *I*_ss_ was exponentially fitted with two time constants *τ*_1_ and *τ*_2_. While *τ*_1_ in the range of 1 ms was compromised by the shutter closing time, *τ*_2_ reflects the slow decay rate constant *k*_2_=1/*τ*_2_. The rate *k*_2_ is linearly dependent on the voltage at different IC pH values (Figure 2 B). Comparing the slopes of linear fits from normalized *I*_ss_ and *k*_2_ versus voltage, no significant differences at any IC pH were found (tests at 0.05 level, not shown). The correlation between *I*_ss_ and *k*_2_ indicates that the currents are kinetically limited by *τ*_2_ as a rate‐limiting step. The influence of the extracellular (EC) pH was investigated by analogous experiments. *I*_ss_ is reversibly reduced due to an EC pH 5.4 (Figure 2 C, Supporting Information, Figure 1 A). This can be explained by a slower pump cycle since the decay time constant *τ*_2_ is significantly decelerated to 20.4±3.0 ms (*n*=5) at pH 5.4 compared to 12.5±0.5 ms at EC pH 7.4 (*n*=5) (also Supporting Information, Figure 1 B). The rate‐limiting step is dependent on the EC pH and interestingly slowed down at a high EC H^+^ concentration. Previously, *τ*_2_ was assigned to the M_2_ (here M_EC_) decay of the photocycle that was suggested to be accompanied by H^+^ uptake from the EC side.^[2]^ The EC pH dependence supports the suggestion that SB reprotonation and H^+^ uptake take place during the rate‐limiting step.

In single turnover experiments the cells were illuminated with a 7 ns light pulse. A typical current signal was observed at 0 mV and IC=EC pH 7.4 (Figure 2 D inset). The rise of the peak current is limited by our time resolution in NG108‐15 cells (≈0.1 ms), but the decay can be fitted with two exponential time constants. For the wild type (WT) *τ*_1_ was 0.45 ms (0 mV, *n*=2) and *τ*_2_ 11 ms (0 mV, *n*=5). The lifetime *τ*_2_ shows a linear voltage dependence (Figure 2 D). Converting time into rate constants brings out the correlation to the continuous illumination measurements in Figure 2 B. Despite robustly *Ns*XeR expressing fluorescent cells, the mutation of the putative acceptor D220N abolished the light‐induced pump currents, which is in agreement with previous findings.^[2]^ D220E resulted in current densities of 1.2±1.0 pA pF^−1^ (at 0 mV, *n*=14) compared to 2.9±2.6 pA pF^−1^ (at 0 mV, *n*=9) for the WT. The linear voltage dependence of stationary photocurrents and decay kinetics was preserved (Supporting Information, Figure 2 A,B), while the rate‐limiting step was significantly slowed down (Supporting Information, Figure 2 B). The single turnover time constant *τ*_2_ for D220E was 34.12±8.9 ms (0 mV, *n*=3), a factor of ≈3 larger than 10.87±0.7 ms (0 mV, *n*=5) for the WT. In UV/Vis absorption spectra *Ns*XeR D220E showed a significantly lowered p*K*_a_ of 8.4 of the SB and a red‐shifted absorption maximum with Δ*λ*_max_=2.6 nm at pH 7.2 (Supporting Information, Figure 3, 4 and Supporting Information, Table 1). The mutation has an influence on the electronic environment close to the retinal bound to the SB and may therefore affect reprotonation or H^+^ uptake kinetics. On the other hand the H^+^ transfer from D220E to the IC may have slowed down, becoming the limiting transition in the photocycle.

Blue light short‐circuits the photocycles of bR and PR by exciting the chromophore in the M intermediate. As a result the retinal reisomerizes and the SB is reprotonated from the primary acceptor at the EC side.[^12^, ^13^, ^14^] Stationary blue light in the presence of the activating light diminishes bR stationary photocurrents while a short laser flash results in fast inwardly directed transient currents.^[13]^ In *Ns*XeR the putative H^+^ acceptor D220 might also serve as the proton donor for the blue light induced reprotonation of the SB. In combination with the photocycle, which shows uniquely two distinct M intermediates, the switch of the accessibility to the cytoplasmic (CP) and the EC side seem to be well defined. With this knowledge and the voltage dependence of the blue light effect as demonstrated below a detailed description of the switch mechanism can be given. Patch‐clamp measurements on *Ns*XeR WT and D220E in response to blue laser ns flashes under saturating activating yellow background illumination (Figure 2 E,F, Supporting Information, Figure 5) showed a complex behavior. The WT blue light induced transients (Figure 2 E) showed a distinct voltage dependence and an inversion of the current direction. At −60 mV the blue flash produced a fast, inwardly directed transient current in addition to the yellow light induced stationary current. At +60 mV the blue light induced current is inverted and shows a quenching effect with fast outward currents. In order to exclude that a blue absorbing ground state (GS) might be responsible for these currents, blue flashes were applied without yellow background light (Supporting Information, Figure 6 A). The resulting time‐resolved currents showed the slower kinetics of a regular single transport cycle and the inwardly directed vectoriality as obtained with yellow light. This proves that only the two M intermediates of the active photocycle are excited by blue light but not the GS or a blue light absorbing GS species with deprotonated SB. This is supported by the results at zero potential difference, where the blue light induced transient current is almost abolished and biphasic. This indicates that the SB reprotonation might occur from both sides with the two M_EC_ and M_CP_ intermediates. Analogous measurements on D220E show stationary currents that are quenched by fast outwardly directed current transients at all membrane potentials (Figure 2 F). However, the observed overshoot reflects simply the shift to higher populations of the M_CP_ in this mutant dominating the reprotonation from the D220 position. In D220E and in the WT at +60 mV blue light produces outwardly directed current transients which have not been observed in quenching experiments with bR, but are expected based on the inverted vectoriality of H^+^ transport by *Ns*XeR. We accordingly hypothesize that the photocycle of *Ns*XeR is likewise short‐circuited by reprotonation of the SB from the primary acceptor. In the case of the putative proton acceptor mutant D220E a voltage‐dependent change of the vectoriality of the transient currents could only be observed without yellow background illumination and at pH 8.4 (Supporting Information, Figure 6 C). We suggest that this is caused by a population of *Ns*XeR with deprotonated SB in the GS due to the lowered p*K*_a_ of the mutant (Supporting Information, Table 1).

**Figure 3:**
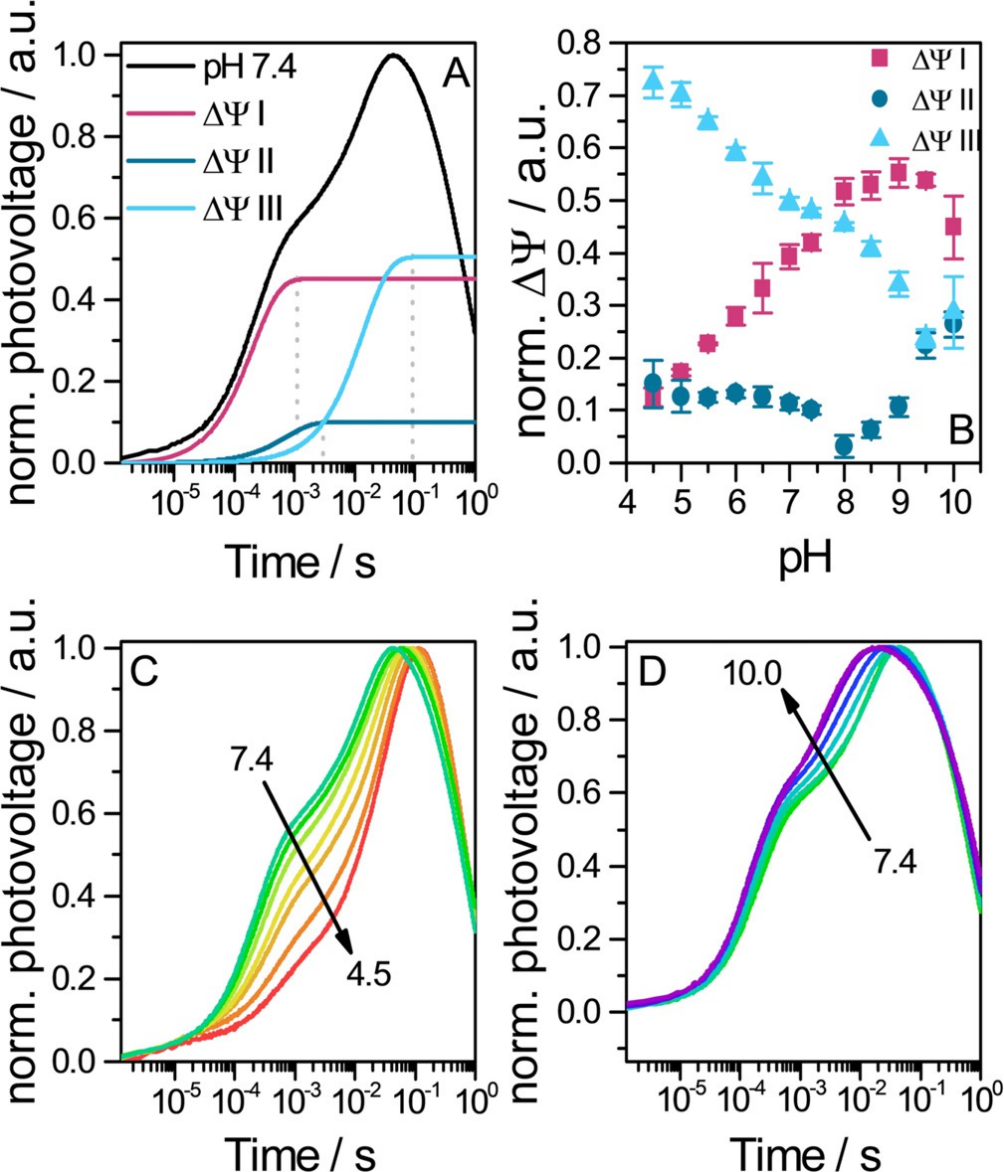
Photovoltages of *Ns*XeR proteoliposomes attached to a BLM. A) Normalized photovoltage at pH 7.4 (black line) and 3 exponential components that were fitted into the build‐up of the signal (plateau starts at dotted line). B) pH dependence of photovoltage build‐up contributions. ΔΨ i (with i=I, II or III) were taken from fits and normalized to the overall voltage generating amplitudes ΔΨ I + ΔΨ II + ΔΨ III (*n*=3). Photovoltage signal upon acidification C) or alkalization D) of the medium.

The black lipid membrane (BLM) system was used to gain a higher time resolution of the electrogenic processes (≈3 μs). Attached to a BLM, *Ns*XeR proteoliposomes caused a voltage signal upon ns illumination (Figure 3 A). Reconstitution yielded a predominant amount of inside‐out oriented protein, which was concluded from the sign of the voltage signal. The kinetic analysis identified 3 successive steps in the signal generation with time constants of approximately 220 μs, 1 ms and 15 ms (pH 7.4). The contributions of the 3 steps accounted for 42 %, 10 % and 48 % of the signal, respectively. The correlation between the electrogenic time constants and flash‐photolysis data^[2]^ (Supporting Information, Figure 7) allowed the assignment of partial charge transfer to transitions in the photocycle. We suggest that *τ*_1_ describes the M_1_ (here M_CP_) formation that is accompanied by SB deprotonation and a H^+^ release to the IC side. *τ*_2_ is assigned to the M_CP_ to M_EC_ (M_2_) transition that presumably includes a switch. The slowest phase *τ*_3_ correlates with the rate limiting step in patch‐clamp experiments that was associated to the M_EC_ decay with a H^+^ uptake from the EC. Starting at pH 7.4, the medium was either acidified (Figure 3 C) or alkalified (Figure 3 D). We observed that the voltage build‐up is delayed in more acidic medium and the maximum is reached at later times. The reason for that is the deceleration of the time constants *τ*_1_ and *τ*_3_ at more acidic pH values (Supporting Information, Figure 8 A,C), whereas *τ*_2_ is widely pH independent above pH 5 (Supporting Information, Figure 8 B). The contributions of each step to the overall photovoltage generation is dependent on the pH value (Figure 3 B). At pH 7.4 steps I and III contribute almost equally. The contribution of step II is comparatively low over a wide pH range. Given that this transition is attributed to the switch, a low contribution makes sense as no protonation/deprotonation processes take place. The shares of steps I and III are strongly pH dependent, but clearly in an opposite way. While only 13 % of the charge is transferred in step I at pH 4.5, step III makes up 73 % of the overall transferred charge. With increasing pH values the share of step I rises and that of step III decreases. An explanation might be that at low pH the SB is deprotonated, but the H^+^ is not further transported to the IC side and stays bound to D220, water or another residue X. When the reprotonation of the SB and the H^+^ uptake occur, the D220 or X bound H^+^ may be simultaneously released with the kinetics of the M_EC_ decay. At higher pH the probability for an immediate H^+^ release from D220 or X may rise and the charge is transferred at an earlier transition in the photocycle.

**Figure 4:**
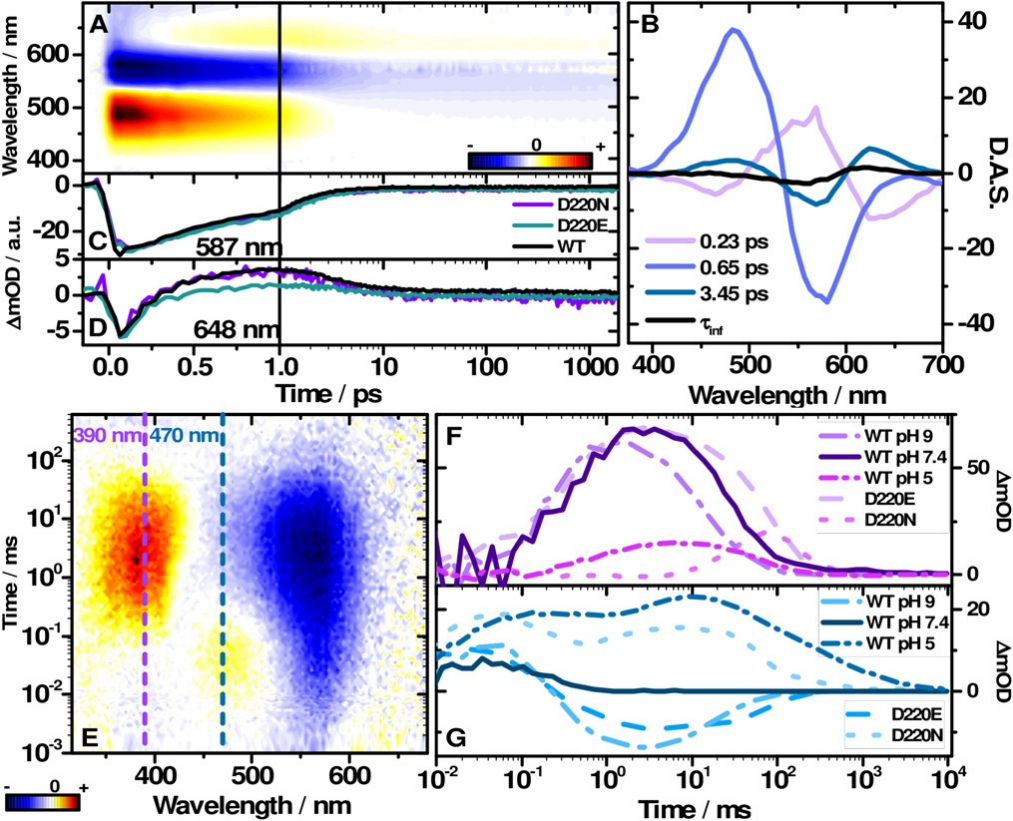
A) Transient absorption map of *Ns*XeR WT and the corresponding decay associated spectra (D.A.S.) in B) with the fitted lifetimes. Transient absorption of the mutants D220N, D220E and the WT detected at C) 587 nm and D) 648 nm. E) Transient map of *Ns*XeR WT. Transient absorption at 390 nm (F), 470 nm (G) of WT pH 7.4, 5 and 9 and the mutants D220E and D220N at pH 7.4.

Femtosecond transient absorption experiments were performed on *Ns*XeR WT at pH 7.4 with an excitation wavelength λ_exc_ of 565 nm to investigate the early photoproduct formation (Figure 4 A). The data was fitted with global lifetime analysis^[15]^ which resulted in 4 lifetime components (see the decay associated spectra (DAS) in Figure 4 B): *τ*_1_=0.23 ps, *τ*_2_=0.65 ps, *τ*_3_=3.45 ps and *τ*_4_=∞ (infinite spectrum at the end of the measured time scale ≈2 ns). Immediately after excitation a broad excited state absorption (ESA) at 480 nm was observed, whereas the GS at 565 nm is depopulated. Additionally, stimulated emission (SE) is visible at 700 nm. The 0.23 ps lifetime component describes the spectral shift dynamics of SE and ESA and the ground state bleach (GSB) signal increases which is a result of the shift of an overlapping ESA band. The SE shifts bathochromically, while the ESA undergoes a hypsochromic shift. This dynamics can be straightforwardly assigned to the relaxation of the excited chromophore from the Franck‐Condon region.[^16^, ^17^, ^18^, ^19^] The ESA and the SE decay with a time constant of 0.65 ps, which is accompanied by some loss of the GSB signal. The GSB amplitude reduces concurrently with the formation of a positive signal at 630 nm. With a time constant of 3.65 ps, this positive band shifts towards 565 nm and partially compensates the GSB (reduced negative amplitude). After this hypsochromic shift, the spectrum remains constant until the end of the recorded time scale and corresponds to the infinite spectrum of the DAS (Figure 4 B). Among the MRs bR shares a similar characteristic absorption change on the ps time scale.[^5^, ^17^, ^19^, ^20^, ^21^] The retinal isomerization is one of the fastest reported photoreactions, occurring with ≈100 fs lifetime.[^22^, ^23^] In *Ns*XeR the photoproduct of the all‐*trans* to 13‐*cis* isomerization can be assigned to the 630 nm band which rises with 0.65 ps. This primary photoproduct is commonly assigned to the vibrationally hot J intermediate.[^17^, ^18^, ^19^, ^20^, ^22^] The 3.45 ps shift of the product absorption band to 618 nm is then linked to the cooling dynamics of the J to the K intermediate. The actual maximum of the K intermediate is located at approximately 596 nm as determined by Gauss function decomposition of the absorption difference spectrum (Supporting Information, Figure 9). Femtosecond transient absorption experiments were also carried out at pH 5 and 9 (Supporting Information, Figure 10) and with the mutants D220N and D220E (Figure 4 C,D). The transient at 587 nm represents the initial depopulation and subsequent partial repopulation of the GS and the rise of the K intermediate. The SE signal and the generation of the J intermediate can be observed at 648 nm. The transients of the WT at pH 5, pH 9 and the mutants show high similarity to the WT at pH7.4 (Supporting Information, Table 2). The early reaction dynamics of the retinal remains basically unaffected.

Flash photolysis experiments of *Ns*XeR by Shevchenko et. al^[2]^ were reproduced in detergent. Here we observed similar lifetimes (Supporting Information, Table 3). After 10 μs the K intermediate is still visible at 618 nm and covers the bleach at 565 nm. The transition to the L intermediate absorbing at 470 nm is associated with an increase of the GSB signal. The fine structure feature of M_CP_, which was previously assigned to a retro‐retinyl configuration,^[2]^ was also present (Figure 4 E). The fine structure disappears in M_EC_ and the absorption becomes spectrally broader (Supporting Information, Figure 12). To gain further insight, flash photolysis experiments for pH 5 and pH 9 and for the mutants D220N and D220E were carried out (Figure 4 F,G).

The transients at 390 nm and 470 nm (Figure 4 F,G) represent the kinetics of the M and L intermediates. Comparison of the experimental data indicates that the M_EC_ intermediate is shifted to earlier times at pH 9, whereas M_CP_ and the L do not change significantly. M_EC_ decays with *τ*_4_=14 ms at pH 9 as compared to *τ*_4_=36 ms at pH 7.4 (Supporting Information, Table 3). The lifetimes of L at pH 9 (*τ*_2_=0.21 ms) and M_CP_ (*τ*_3_=3.4 ms) are still similar to pH 7.4 (L: *τ*_2_=0.29 ms, M_CP_: *τ*_3_=2.5 ms). This indicates that only the reprotonation step is affected at pH 9. Interestingly, at pH 5 the fine structure at 390 nm is missing, and the transient can be fitted with only one component *τ*_4_. In this case a distinction between M_CP_ and M_EC_ is not possible. The M intermediate is temporally delayed with *τ*_4_=48 ms. The signal associated with L is significantly extended over time (Figure 4 G). Moreover, in the transient absorption at 470 nm two distinct amplitudes occur for the L intermediate, whereas the M intermediate absorption at 390 nm is drastically weaker compared to pH 7.4. Yet, the lifetimes of the K intermediate (*τ*_1_=10 μs) and the first L component (*τ*_2_=0.39 ms) at low pH do not differ much from the lifetimes at pH 7.4. However, the second L contribution decays with the same lifetime as the M intermediate.

The kinetics of all intermediates except K are affected (Figure 4 F,G, Supporting Information, Table 3) in the mutant D220E. M_EC_ is more than twice longer as (*τ*_4_=80.86 ms) compared to the WT (*τ*_4_=35.83 ms). While the time constant of L is slightly longer (*τ*_2_=0.44 ms), the decay of M_CP_ is more than threefold longer. Although, H^+^ pumping and the intermediates are maintained in D220E, the protonation process is significantly slower. Exchanging D220 with an E preserves the acidic function, yet extends the residue chain by a CH_2_ unit. This altered steric requirement might change distance and accessibility for H^+^ transfer partners and lead to delayed photocycle dynamics. In D220N no H^+^ pumping was observed^[2]^ indicating loss of functionality. However, photoinduced retinal isomerization and photocycle intermediates were observed (Figure 4 C,F,G). Compared to D220E, formation of M_EC_ of D220N is even further delayed to *τ*_4_=152.84 ms. Despite the fact that H^+^ pumping was not observed,^[2]^ the L and the M states still occur: Whereas M shows a low transient amplitude, L is more prominent and extends to the end of the photocycle. This behaviour is comparable to the L intermediate dynamics in the WT photocycle at pH 5. A similar effect at lower pH was also observed for bR D96N and D115N/D96N,^[24]^ where L persists longer at low pH and coexists with M.^[24]^ Due to the strong temporal overlap of L and M, a higher back reaction rate of M to L was suggested.^[24]^ Yet, in the case of the *Ns*XeR D220N the M intermediate accumulates after the decay of L and a distinction of two M intermediates is not necessary to describe the photocycle dynamics. In bR the transition from M_CP_ to M_EC_ was linked to the so‐called switch mechanism,[^25^, ^26^] a fundamental model describing the unidirectional pumping pathway of MRs. Essentially, the isomerization‐switch‐transfer (IST) model[^26^, ^27^] describes that the H^+^ acceptor is separated from the SB after deprotonation to prevent reprotonation from the same side. The switch is controlled through conformational changes and the change of the p*K*_a_ of the acceptor.^[26]^ In analogy to bR, M_CP_ of *Ns*XeR with its fine structure should be the deprotonated species before the switch. The three peaked absorption band can be a result of an enforced retinal structure. Enforcing planarity of the π‐system of the retinal through the amino acid residues within the binding pocket, results in a retro‐retinyl like spectrum.^[28]^ The loss of the fine structure with the M_CP_→M_EC_ transition can be considered a direct indication for the switch since the absorption maximum of M_EC_ remains at 390 nm (Figure 4 E). In the case of D220N no M_CP_ formation could be observed, that is, either no switching occurs at all or at another part in the photocycle. Interestingly, the time constants for the L_CP_ to L_EC_ transition of pH 5 (*τ*_3_=3.08 ms) and D220N (*τ*_2_=2.20 ms) match the M_CP_ to M_EC_ transition of WT pH 7.4 (*τ*_3_=2.49 ms). It has been shown that the protonation switch is thermodynamically independent of the retinal intermediates,[^26^, ^29^, ^30^, ^31^] that is, the distinct chromophore conformations do not have to be coupled with the changes within the protein. Consequently, if the two L intermediates result from the presence of a pre‐switched and a post‐switched species, this would explain why only M_EC_ can be observed for pH 5 and D220N.

**Figure 5:**
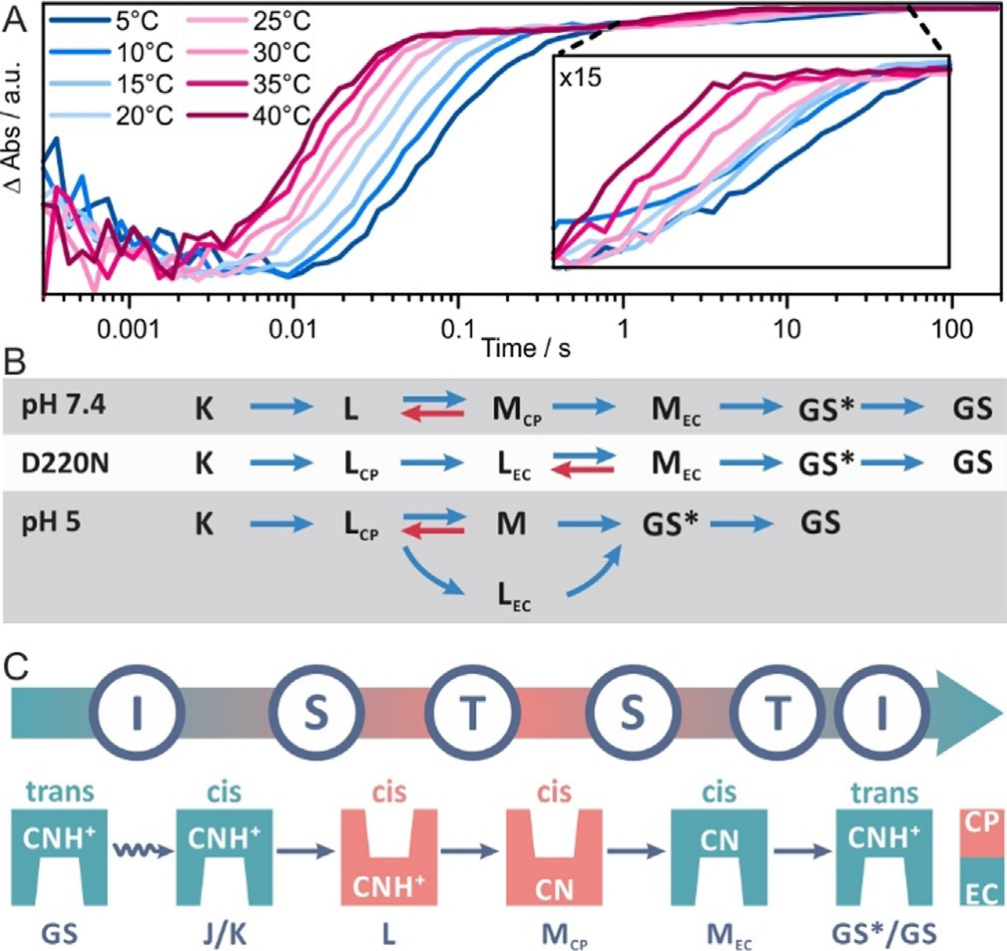
A) Temperature dependence of the *Ns*XeR photocycle demonstrated at the GSB at 565 nm. The photocycle extends clearly beyond 1 s. B) Kinetic models that best describe the experimental data (prior to selecting these models a wide range of kinetic models, including purely sequential, branched and back rate including models between all intermediates, were tested). C) The IST model applied to the photocycle of *Ns*XeR (see Supporting Information, Figure 14 for comparison with bR).

The temperature dependence of the WT *Ns*XeR photocycle in the range from 5 °C to 40 °C was investigated (Figure 5 A). Overall, the temperature decrease leads to slower transient kinetics and vice versa. We observe a second slow recovery step of the GSB (Figure 5 A) which was not previously reported. At 20 °C the last component decays with 8.6 s and is clearly temperature dependent. A similar residual bleach has been observed for *Po*XeR[^4^, ^32^] which was assigned to a metastable 13‐*cis* state. Therefore, in the case of the photocycle of *Ns*XeR the very last component might be an additional intermediate similar to 13‐*cis Po*XeR. This intermediate either has a very low extinction coefficient or its absorption maximum is very close to the GS. In the latter case the intermediate could be a prearranged GS similar to the P_4_ state of ChR2.^[33]^ Therefore, this intermediate is called GS*. The kinetics of the temperature dependent transients follows an Arrhenius dependence (Supporting Information, Figure 13). The slopes of the Arrhenius plots for the different components are very similar and yield an activation barrier of 40 kJ mol^−1^ for most of the kinetic steps except for the second step (assigned to the L→M transition) with *E*_a_=30 kJ mol^−1^ and the third step (assigned to M_CP_→M_EC_ transition) with *E*_a_=18 kJ mol^−1^.

All the flash photolysis experiments were subjected to an extensive kinetic fitting procedure to find the most appropriate model that provides a mechanistic picture of the *Ns*XeR photocycle reactions. The model fitting was performed using global target analysis where all experimental datasets were fitted simultaneously with kinetic models that shared certain rates to reduce the degrees of freedom. Furthermore, the Arrhenius dependence of the rates was introduced as a condition to limit the number of possible model solutions. The analysis resulted in the models shown in Figure 5 C. The flash photolysis data from the experiments at neutral pH is best described by a sequential model that includes an equilibrium between the L and the M_CP_ intermediates. Interestingly, the rate of the M_CP_→L back reaction (0.94 s^−1^) is higher compared to the M_CP_→M_EC_ rate (0.68 s^−1^), that is, the L to M_CP_ transition (2.4 s^−1^) might be a bottleneck in the photocycle. The population of L during the photocycle strongly overlaps with M_CP_ (Supporting Information, Figure 11). Therefore, M_CP_ is never highly populated, it either reverts back to L or converts to M_EC_. The photocycle ends with the decay of the GS* intermediate (1.16×10^−4^ s^−1^). The significantly altered flash photolysis data at pH 5 and for the D220N mutant required modified kinetic models. An additional intermediate called L_EC_ had to be introduced, which accounts for the second L spectral signature at 470 nm persisting at longer timescales. A sequential kinetic model was found for the photodynamics of D220N in which L_CP_ directly converts to L_EC_ without a back rate. This L_CP_→L_EC_ transition is possibly induced by the switch which is kinetically independent from the retinal dynamics and can be irreversible on this time scale.^[29]^ The following intermediate is referred to as M_EC_ and shows similar spectral characteristics as the M_EC_ intermediate of the WT, which also occurs after the switch. Our results indicate that the M_EC_ intermediate is in equilibrium with L_EC_ with a relatively slow back rate (0.008 s^−1^), which explains its low transient population (Supporting Information, Figure 11) as compared to M_EC_ of the WT. In effect, the L_EC_ intermediate shows a high transient population and therefore the L_EC_→M_EC_ transition appears to be a bottleneck in the photocycle of the D220N mutant. A modified kinetic model Scheme was also required to analyze the flash photolysis data at pH 5 (Figure 4 F,G). The photocycle is branched and the decay of L_CP_ results in the formation of both the L_EC_ and an M intermediate (M_CP_ and M_EC_ intermediates could not be spectrally distinguished and therefore the deprotonated intermediate is only referred to as M). Further, we found that L_CP_ is in equilibrium with M (M→L_CP_ back rate of 0.56 s^−1^). Interestingly, decay of both the M and the L_EC_ intermediates can lead to the formation of the GS* intermediate with an M→GS* rate (0.34 s^−1^) being more than 20 fold higher than the L_EC_→GS* rate (0.014 s^−1^). This behaviour is reflected in the corresponding populations (Supporting Information, Figure 11) with the population of GS* extending along the time scale and overlapping with populations of L_EC_ and M. The higher amplitude for GS* at 10 ms compared to L_EC_ also indicates a higher M→GS* rate. Overall the kinetic model reproduces the experimentally observed spectral dynamics very well.

## Discussion

In electrophysiological and spectroscopic measurements the acceleration of the M_EC_ decay at higher pH values could be observed (Supporting Information, Figure 7). The pH dependence excludes a direct reprotonation of the SB from the EC bulk during this transition since a deceleration of the decay would be expected in this case.^[34]^ The SB is more likely reprotonated from a donor group that was suggested to be D76.^[2]^ The UV/Vis absorption spectrum of *Ns*XeR WT is hardly affected by pH changes (Supporting Information, Figure 4), which indicates that the counterion D76 stays deprotonated in the GS. If D76 acts as a donor, the H^+^ is bound transiently during the photocycle. The reprotonation of the SB might be dependent on the protonation state of a group near the active center that might block the H^+^ transfer at lower pH. An influence of the protonation state of D220 in the IC half can be ruled out, since only the EC pH caused changes in reprotonation kinetics. The acceleration of the SB reprotonation at higher pH values was also observed for *Po*XeR^[4]^ and ASR.^[35]^ This might be a feature typical for this family of MR and its homologs. At low pH the photocycle model from the spectroscopic data shows a branched reaction after L_CP_ with a decay path that does not include a M like intermediate (Figure 5 C). In that case it is assumed that no H^+^ is transported. The reduction of the stationary photocurrent at IC pH 5 can mainly be explained by the slower kinetics (Figure 2 C) as shown by the electrophysiological data. Assuming that part of the excited proteins cycle without pumping due to the split photocycle model, this part would have to be compensated in the patch‐clamp measurements. This could be the case, if for example the percentage of excited *Ns*XeR is also dependent on the IC pH.

Mutational studies on putative crucial side chains in the H^+^ transfer pathway provide insights into the overall mechanism. The measurements on D220E/N revealed the central importance of this group for the inward H^+^‐pump mechanism. Neutralization of the side chain in D220N impaired photocurrents and revealed a significantly delayed and reduced appearance of a M intermediate (Supporting Information, Figure 7). The results confirm that D220 acts as the acceptor to the SB H^+^. The D220E mutation lowers the SB p*K*_a_ from 9.6 to 8.4 which shows its influence on the electronic environment of the active center. The overall pump cycle is slowed down, which might be caused by the above mentioned effect that alters the reprotonation kinetics from the donor. Alternatively, another step might have become rate limiting. The probably higher H^+^ affinity of Glu compared to Asp might for example retard the release of a H^+^ to the IC solution.

Spectral indicators for the switch are the M_CP_→M_EC_ and L_CP_→L_EC_ transitions. They occur at the same time scale which demonstrates that the switching process is kinetically uncoupled from the photointermediates of the photocycle. For the WT, the loss of the M_CP_ fine structure at the transition to the M_EC_ represents the steric influence of the protein binding pocket to the retinal conformation during the switching process. For the non‐pumping D220N the M_CP_→ M_EC_ transition is not visible anymore due to the missing acceptor group. For the late occurring M_EC_ we suggest that the SB is deprotonated and reprotonated from the same side. However, the L intermediate becomes more prominent with two populations caused by the switch.

**Figure 6:**
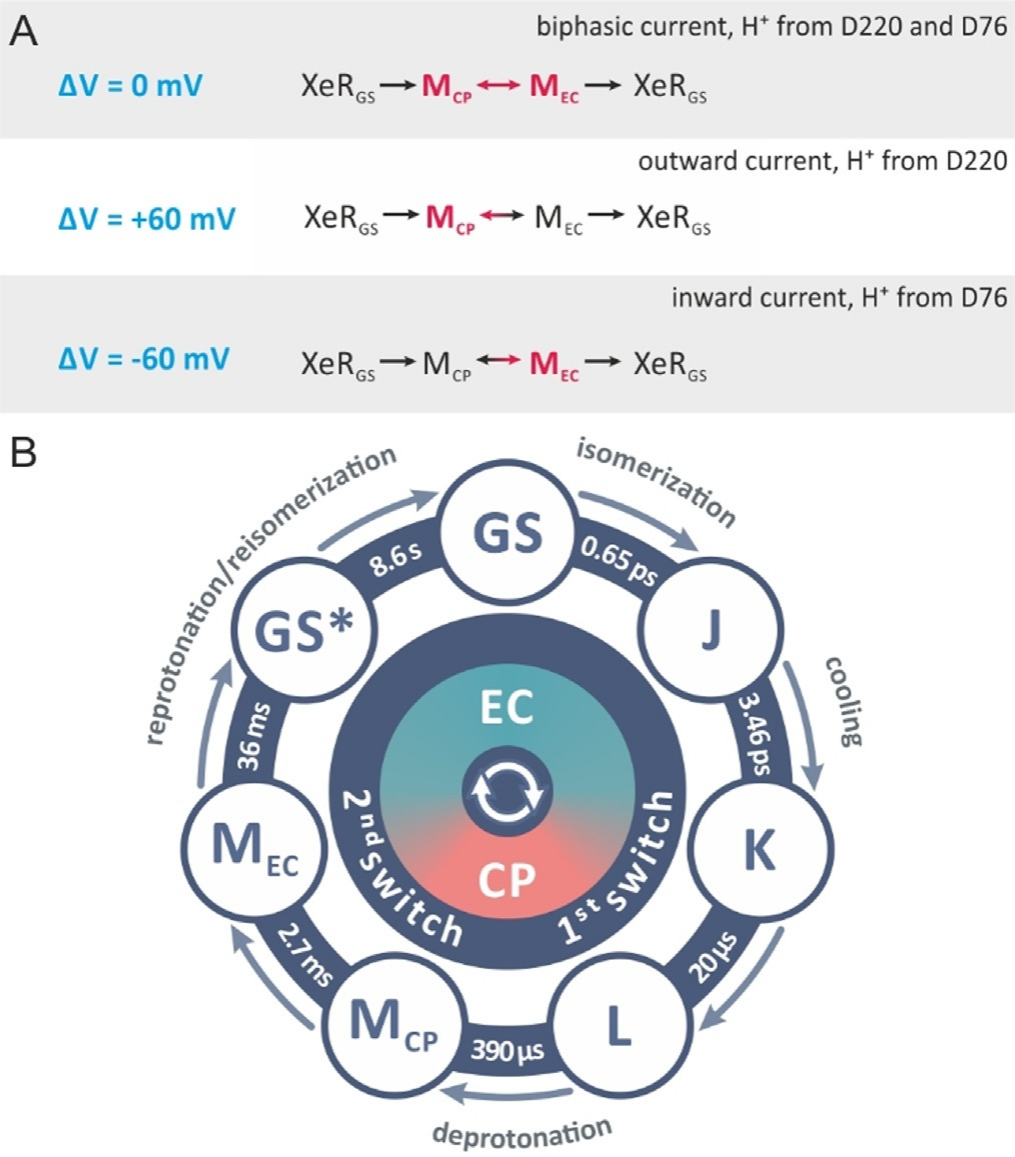
A) Scheme for the blue light induced transient currents in *Ns*XeR. At 0 mV M_CP_ and M_EC_ are nearly equally populated. At 60 mV and −60 mV the equilibrium is shifted to M_CP_ and M_EC_, respectively. B) Photocycle model for the WT *Ns*XeR.

To our knowledge, the voltage dependent variability of the blue light activated transient currents in *Ns*XeR was not reported for another MR before. In *Ns*XeR two blue absorbing intermediates M_CP_ and M_EC_ can accumulate during continuous yellow background illumination. It was suggested that during the M_CP_ to M_EC_ transition the accessibility of the SB switches from the IC to the EC.^[2]^ For this we propose a simplified model for the voltage dependent switch and the accompanying blue light induced transient currents. In Figure 6 the photocycle of *Ns*XeR can be formulated for the experimental situation in Figure 2 E. At 0 mV the blue light effect is very small and biphasic. This means the distribution between M_CP_ and M_EC_ is almost equal so that the contribution of the blue light induced transient current is nearly canceled. Here we assume that in M_CP_ the light induced reprotonation occurs from the cytoplasmic side, probably from the H48/D220 pair,^[2]^ yielding a small but distinct outward current and M_EC_ reprotonates from the extracellular side from D76 accompanied by an inward current. An explanation which is based on a fast equilibrium between M_CP_ and M_EC_ and blue light induced protonation reactions is further supported by the low activation barrier of the M_CP_/M_EC_ transition (Supporting Information, Figure 13) and by the surprising variability of the sign of the transient currents at the different applied potentials (Figure 2 E, Supporting Information, Figure 5). The effect of the applied potential on the concentration ratio between M_CP_ and M_EC_ can be probed by the application of the short blue light laser pulses. The application of a positive potential results in the slower decay of M_EC_, which is reflected in the decreased yellow light stimulated stationary photocurrent (Figure 2 E, Supporting Information, Figure 5). From the polarity of the charge transfer during the M_CP_/M_EC_ transition^[2]^ (Figure 3) we suggest that a positive membrane potential decelerates the M_CP_/M_EC_ transition and accelerates the M_EC_/M_CP_ transition. The slower decay of M_EC_ and the potential dependence of *k*_MCP/MEC_ and *k*_MEC/MCP_ shift the equilibrium between M_CP_ and M_EC_ in direction of M_CP_. The blue flash leads to a large outwardly directed transient current (Figure 2 E, Supporting Information, Figure 5), which indicates that the flash‐induced isomerization of M_CP_ probably results in the reprotonation from D220 and not in the switch from M_CP_ to M_EC_. The opposite occurs at negative potentials, where the pump current is increased and the M_CP_/M_EC_ ratio is shifted to M_EC_, which then is reprotonated from D76. The mutation in D220E supports the view in the Scheme (Figure 6 A), because the mutation slows down the rate limiting step in the photocycle, the M_EC_ decay, which results in an increase of M_CP_, so that the blue light induced transient current is outwardly directed (Figure 2 F). The biphasic behavior at 0 mV and −60 mV indicates that at these potentials a significant part of the reprotonation occurs on M_EC_ (Figure 2 F).

Our study represents a joint spectroscopic and electrophysiological characterization of the inward H^+^ pump mechanism of *Ns*XeR. *Ns*XeR has the potential to be a powerful optogenetic tool since compared to the well‐established ChR2 it allows for cation gradient independent cell depolarization. Our findings show the IC pH independence of the pump turnover and allow the detailed description of the kinetically independent molecular switch that determines the accessibility of the SB for protonation and deprotonation events and by this the vectoriality of the pump. Furthermore, our data can be also used for better planning of time‐resolved crystallographic experiments at free‐electron lasers aimed to determine details of molecular mechanisms of inward proton pumping by XeRs.

## Conflict of interest

The authors declare no conflict of interest.

## Supporting information

As a service to our authors and readers, this journal provides supporting information supplied by the authors. Such materials are peer reviewed and may be re‐organized for online delivery, but are not copy‐edited or typeset. Technical support issues arising from supporting information (other than missing files) should be addressed to the authors.

Supporting Information
