# Supplementary material for: The Voltage Dependent Sidedness of the Reprotonation of the Retinal Schiff Base Determines the Unique Inward Pumping of Xenorhodopsin
Source: Angew Chem Int Ed Engl. 2021 Sep 15;60(42):23010–7. doi: 10.1002/anie.202103882 (PMC8518763; doi:10.1002/anie.202103882)
Supplement: Supplementary file 1 — Supporting Information [file ANIE-60-23010-s001.pdf]

Angewandte Chemie
Eine Zeitschrift der Gesellschaft Deutscher Chemiker

Supporting Information

The Voltage Dependent Sidedness of the Reprotonation of the Retinal Schiff Base Determines the Unique Inward Pumping of Xenorhodopsin

Juliane Weissbecker[+], Chokri Boumrifak[+], Maximilian Breyer, Tristan Wießalla, Vitaly Shevchenko, Thomas Mager, Chavdar Slavov, Alexey Alekseev, Kirill Kovalev, Valentin Gordeliy, Ernst Bamberg, and Josef Wachtveitl**

anie_202103882_sm_miscellaneous_information.pdf

Supporting Information

Methods

Sample preparation. For physiological conditions a 100 mM phosphate buffer at pH 7.4 with 100 mM NaCl and 0.2% DDM was used in the spectroscopic experiments. The pH dependencies were investigated in a titration buffer consisting of 100 mM NaCl, 20 mM HEPES, 20 mM H_3BO_3, 20 mM $C_6H_8O_7$ and 0.05% of the detergent n-dodecyl-β-D-maltoside (DDM).

Flash photolysis. The photodynamics in the µs-s time scale was recorded with two different setups. A broadband flash photolysis setup, was applied to monitor the photocycle over the whole absorption spectrum, which was detected via an ICCD camera. This setup was described in detail by Urmann et al. [1]. A second setup was used to detect transients at specific wavelengths with a photodiode. The signal was recorded by a Tektronix oscilloscope which enabled scans beyond minutes. The excitation energy of the pump pulse at 565 nm was set to 2.5 mJ.

Ultrafast Experiment. The setup has been described in detail by Slavov et al. [2]. A Clark laser of the MXR CPA-iSeries with a repetition rate of 1 kHz, a central wavelength of 775 nm and a pulse width of 150 fs was used. To generate the 565 nm pump pulse a home-built NOPA was applied. Additionally, the time resolution was adjusted with a prism compressor to 60 fs. The energy was set to 30 nJ per pulse. The probing white light (300-700 nm) was generated in CaF_2.

Data analysis. The time resolved data were analyzed by using OPTIMUS [3]. Global life time analysis and life time density maps were calculated to gain the life times of the occurring intermediates.

Absorption spectroscopy. *Ns*XeR was expressed in E. coli cells and purified according to a protocol that was described previously [4]. Absorption spectra at different pH values were taken using a Varian Cary 50 Bio UV-Visible spectrophotometer. *Ns*XeR protein was solubilized in a buffer consisting of 100 mM NaCl, 20 mM Hepes, 20 mM H_3BO_3, 20 mM $C_6H_8O_7$ and 0.05% of the detergent DDM. The measurements were performed under room light at approximately 25°C. The buffer solution was adjusted to different pH values by adding small amounts of 1 N NaOH or HCl. The reversibility of spectral changes was checked by titrating back to the original pH. For the data analysis, the relative absorption changes at certain wavelengths were plotted against the pH. The data was fitted using the logistic fit function

$$\Delta Abs = \frac{A}{1 + 10^{n(pKa - pH)}}$$

with A representing the amplitude of the absorption changes and n the Hill coefficient. The fits yielded values of n < 1.

Expression of *Ns*XeR in mammalian cells. NG 108-15 cells were transiently transfected with a pcDNA3.1(-) vector that carried the human codon-optimized *Ns*XeR gene together with a membrane trafficking signal, a self-cleaving 2A peptide and the red fluorescent protein Katushka [5]. The transfection reagents Lipofectamine 2000 or LTX from Thermo Fisher Scientific were used according to the manual instructions. Transfected cells were incubated for two days at 5% CO_2 and 37°C.

Electrophysiological recordings. For patch-clamp recordings on *Ns*XeR expressing NG108-15 cells an Axon Instruments Axopatch 200B amplifier and a Digidata 1440A digitizer were used. Thin-walled borosilicate glass capillaries (GB150F-8P, Science Products) and a P-1000 Sutter Instruments micropipette puller were used to prepare patch pipettes with resistances between 2 to 5 MΩ.
Whole-cell patch-clamp recordings were performed at various intra- and extracellular pH values. Pipette solutions consisted of 110 mM NaCl, 10 mM EGTA, 2 mM $MgCl_2$ and 10 mM buffer. The composition of the bath solutions was 140 mM NaCl, 2 mM $MgCl_2$, 2 mM $CaCl_2$ and 10 mM buffer. The buffer for pipette and bath solution was

chosen according to the desired pH. Solutions at pH 5.4 used $C_6H_8O_7$ for a buffer. MES was used for pH 6.0 and 6.4, whereas HEPES was used for pH 7.4. Basic solutions at pH 8.4 were buffered with Tris.
Light-triggered currents were measured upon continuous illumination with a diode-pumped solid state laser (MLL-FN-561 by Photontec) at 561 nm. Measurements were performed at saturating light-intensities of at least 23 mW/mm^2. Nanosecond light pulses were created with an Opolette 355 tunable laser by Opotek.
Measurements with continuous illumination at intracellular pH 7.4 and different extracellular pH values were preceded and followed by measurements at extracellular pH 7.4. A run-down correction was calculated as follows:

$$I_{\Delta pH,corrected} = \left(\frac{1 + \frac{I_{7.4,after}}{I_{7.4,before}}}{2} \right)^{-1} I_{\Delta pH}$$

with I_7.4 and I_∆pH representing the steady-state photocurrents at symmetrical IC and EC pH 7.4 and at pH gradients, respectively.
Patch-clamp signals were filtered at 5 kHz (for continuous illumination measurements) or at 10 kHz (for nanosecond pulse illumination) and were recorded at more than twice its filter-frequency. The data were acquired and analyzed using Axon pCLAMP 10.4 from Molecular Devices and Origin 2018b software.

Reconstitution of *Ns*XeR into Liposomes. E. coli polar lipids dissolved in $CHCl_3$ by Avanti were dried under nitrogen atmosphere by rotary evaporation for 30 min. The dried lipid film was hydrated with a solution containing 100 mM NaCl, 20 mM HEPES, 20 mM H_3BO_3 and 20 mM $C_6H_8O_7$ at pH 7.4. The lipid suspension was vortexed and agitated during a hydration time of about 30 min. 1.5% tris-cholate was added and the solution was vortexed and left to rest until it appeared opalescent. Upon addition of purified *Ns*XeR protein, the mixture was incubated on ice for 10 min. The detergent was removed by overnight agitating with Bio Rad's Bio-Beads SM-2 at 4°C. The usual preparation volume was 600 µl with a lipid concentration of 10 mg/ml and 10 µM protein yielding a protein/lipid ratio of 1:40 (w/w).

BLM measurements. Light-induced photovoltages of *Ns*XeR were measured using proteoliposomes that adsorbed to a black lipid membrane of 1 mm^2. Experiments were carried out in a Teflon cell with two chambers which were filled with 1.3 ml of the sodium chloride solution that contained 100 mM NaCl, 20 mM HEPES, 20 mM H_3BO_3 and 20 mM $C_6H_8O_7$ at pH 7.4. The membrane-forming solution contained 1.5% (w/v) 1,2-diphytanoyl-sn-glycero-3-phosphocholine (DPPC) by Avanti and 1:60 (w/w) octadecylamine (Fluka 74750) to DPPC dissolved in decane. The capacitance and conductivity of the membranes were around 2-2.5 nF and 10-100 pS, respectively. *Ns*XeR containing proteoliposomes were sonicated for 1 min. Afterwards 15 µl were added to the chamber averted to the light source. After at least 1 h 5 µM monensin was added to the medium to abolish possible proton gradients between interior and exterior of the vesicles. Measurements were started earliest 30 min later. The chambers of the cell were connected to an external measuring circuit via salt bridges and Ag/AgCl electrodes. Electrically sensitive parts of the set-up were located in a Faraday cage. The photovoltage was amplified by a factor of 66 before the signal was coupled into a SR 560 low-noise preamplifier by Stanford Research Systems. The signal was filtered with 300 kHz cut-off frequency and the amplification was usually set to 1. The signal was written using a Pico Technology ADC-212 digital oscilloscope with the associated PicoScope6 software. Photovoltages were measured with two different sampling intervals: 1.28 µs and 81.92 µs. The overall time resolution for the voltage measurements yielded a 10-90 rise time of 3 µs. For the measurements 556 nm light-pulses of ≥ 10 ns were applied using a XeCl excimer laser-pumped dye laser (Lambda Physik COMPex) with Rhodamine 6G. The electrolyte solution was adjusted to different pH values by adding small amounts of 1N NaCl or HCl. Measurements were repeated four times at each pH value to make sure that it was stable. After the titration experiments the final pH was checked. The maximal deviation from the intended value was 0.13. The data was processed and analyzed using Origin 2018b software. In order to cover a sufficiently long time range a LabTalk script (by C. Bamann) was used that reduced the data density with increasing time and combined the two data sets of different sampling intervals. The photovoltage signal was fitted with a sum of exponential functions including the relaxation of the signal with τ_{sys}:

$$\psi(t) = \psi_I e^{-t/\tau_1} + \psi_{II} e^{-t/\tau_2} + \psi_{III} e^{-t/\tau_3} + \psi_{sys} e^{-t/\tau_{sys}} + c$$

Between pH 4.5 and 7.4 the signal relaxed with a single slow time constant of 0.6 to 1 s. At pH values of 8.0 and higher, a second decay time constant of 50 ms to 90 ms had to be introduced. An explanation for this second relaxation time constant might be that different pH values cause different degrees of coverage of the membrane.

The access to the medium would vary and result in different time constants. Furthermore, the fast proton transport leads to a voltage as well as a proton gradient, which both need to dissipate.

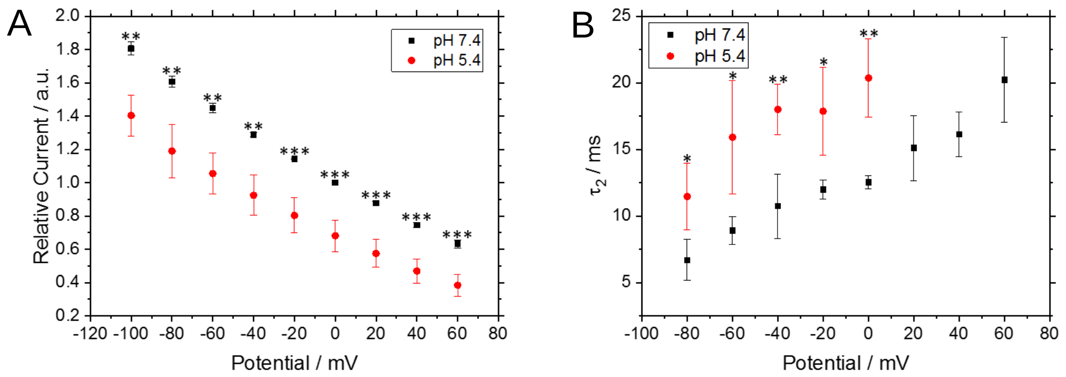

Figure S1. Voltage dependence of relative stationary currents (A) and slow decay time constant τ2 (B) from continuous illumination measurements with 561 nm at extracellular pH 7.4 and 5.4. The intracellular pH was kept at 7.4. (A) The stationary photocurrents were rundown corrected and normalized to the current at 0 mV, pH 7.4. The differences were significant with: * $p < 0.01$, ** $p < 10-3$ and *** $p < 10-4$ (with $n = 5$).

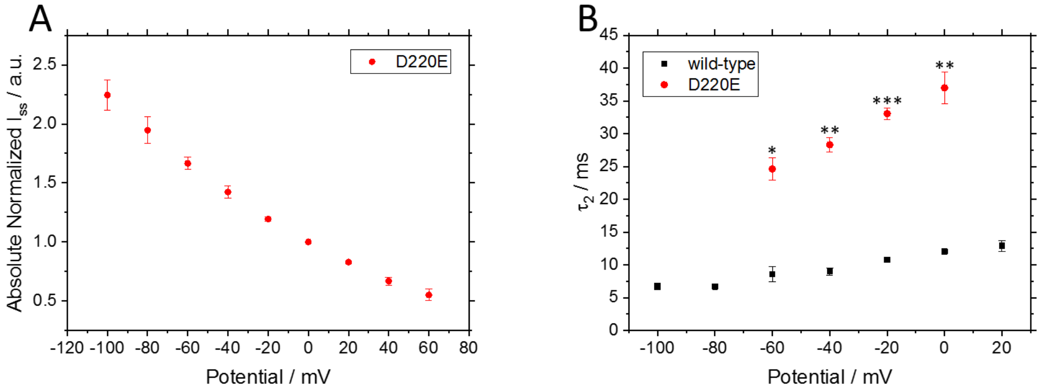

Figure S2. Voltage dependence of absolute normalized stationary currents (A) and slow decay time constant τ_2 (B) from continuous illumination measurements with 561 nm at pH 7.4 on *Ns*XeR D220E. (A) The stationary photocurrents of *Ns*XeR D220E were normalized to the current at 0 mV and are given as absolute values (n = 10). (B) The voltage dependence of the slow decay time constant τ_2 for the mutant D220E (n = 3) compared to the wild type (n = 3). The differences in τ_2 between the wild type and D220E were significant with: * p < 10-3, ** p < 10-4 and *** p < 10-5.

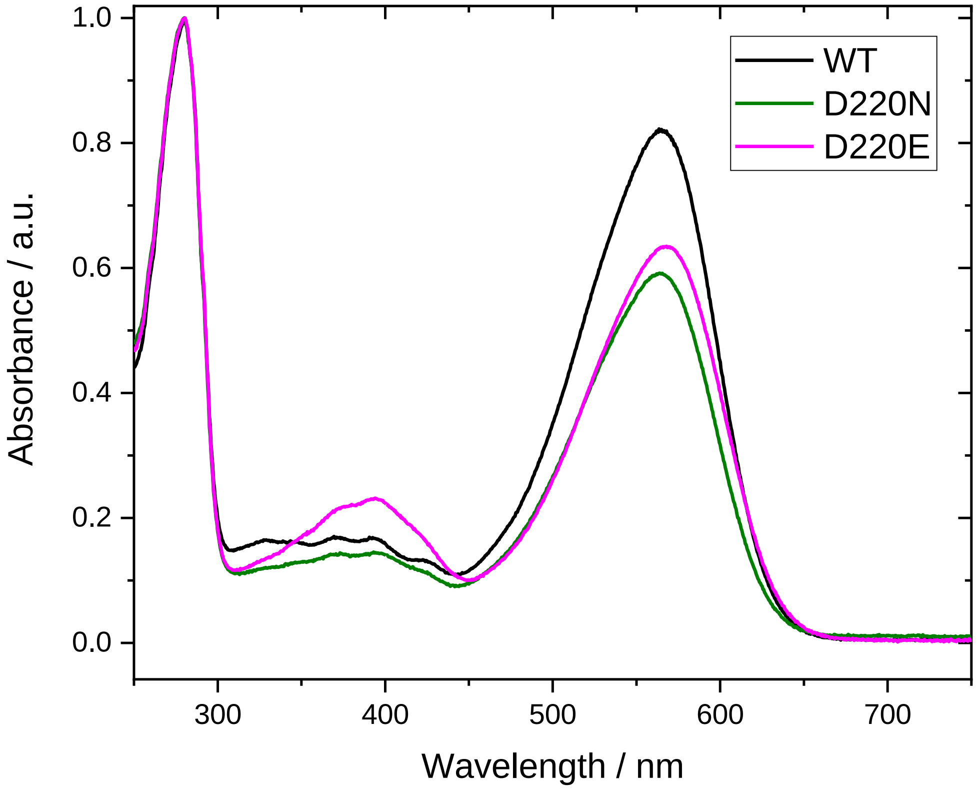

Figure S3. UV-Vis spectroscopy of *Ns*XeR. (A) Absorption spectra of *Ns*XeR wild type and mutants D220N and D220E solubilized in DDM at pH 7.2 relative to absorbance at 280 nm.

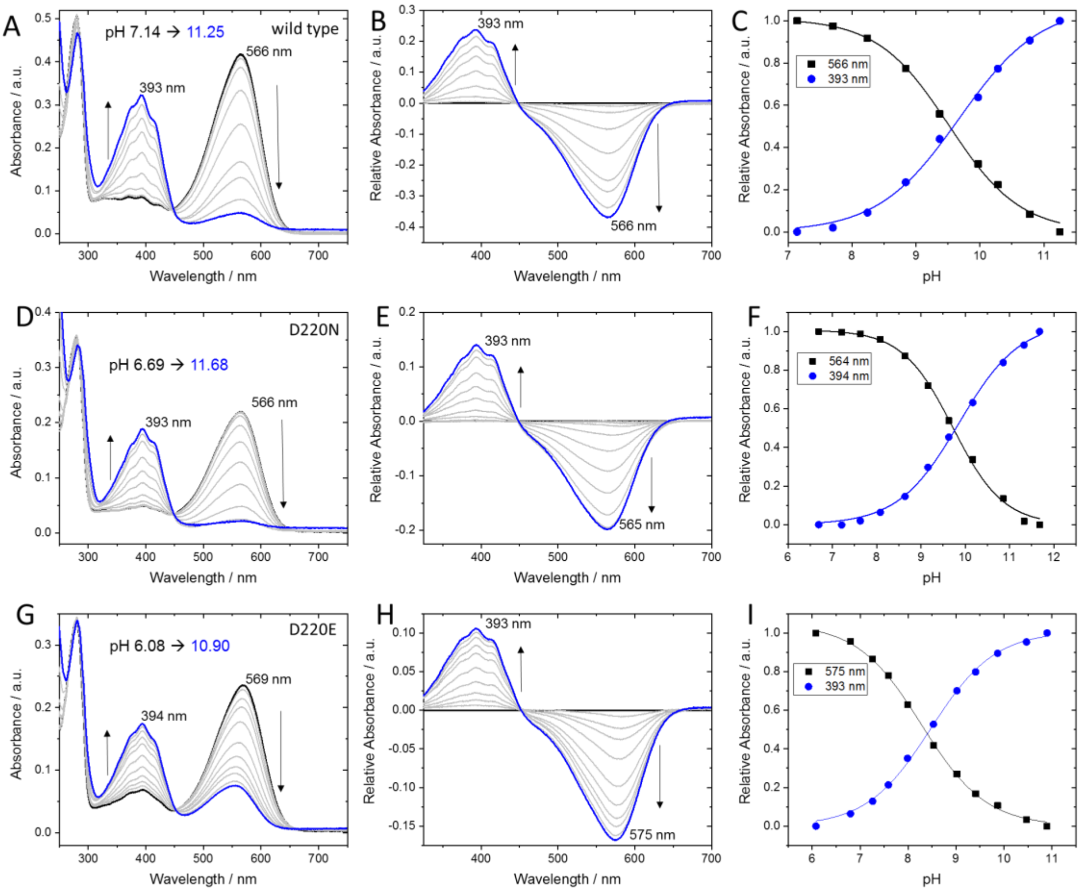

Figure S4. UV-Vis spectroscopy of *Ns*XeR wild type (A-C), D220N (D-F) and D220E (G-I). (A, D, G) Spectral changes induced by pH titrations. (B, E, H) Difference spectra at different pH values. The spectrum at the initial pH was subtracted from each spectrum. (C, F, I) Relative absorbance change at peak wavelengths from B, E and H versus pH. The apparent pKa of the retinal Schiff base was identified by fitting the data with a logistic function with n<1.

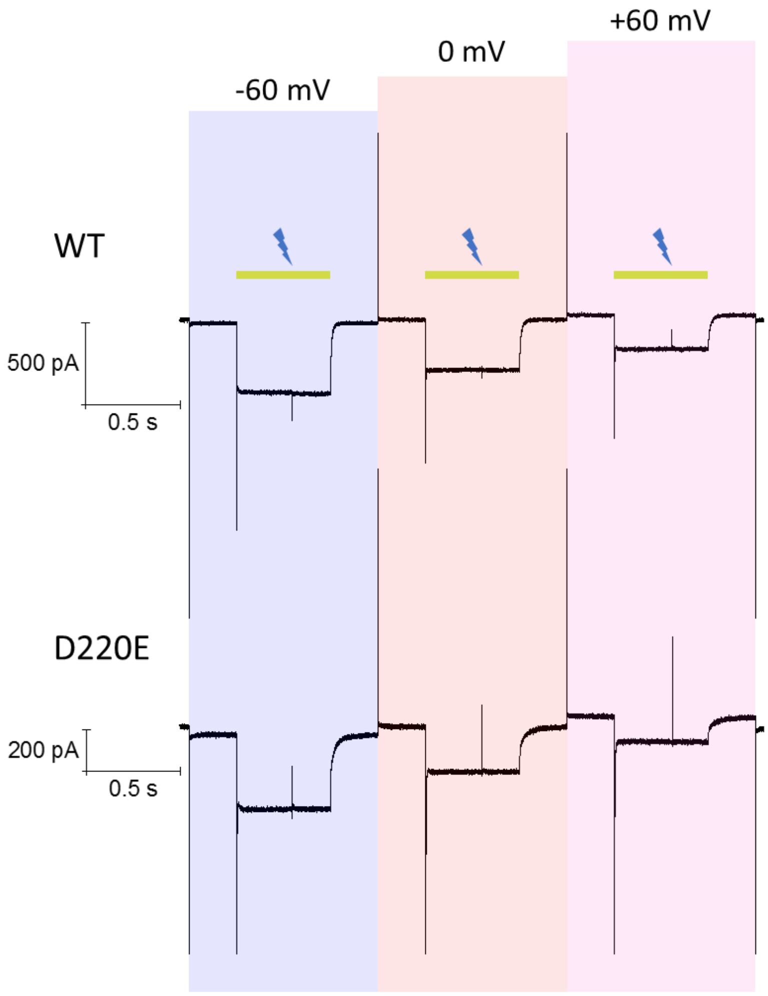

Figure S5. Raw data and illumination protocol of patch-clamp measurements with continuous 561 nm illumination and laser flashes at 355 nm.

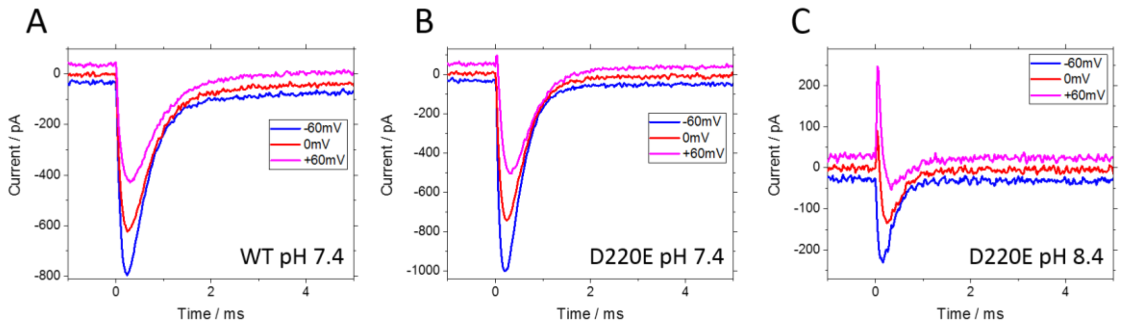

Figure S6. Transient photocurrents of *Ns*XeR expressing NG105-18 cells. Wild type (A) and mutant D220E (B and C) current transients were measured in response to 355 nm laser flashes without background illumination. The pH was symmetrically set to 7.4 (A and B) or 8.4 (C).

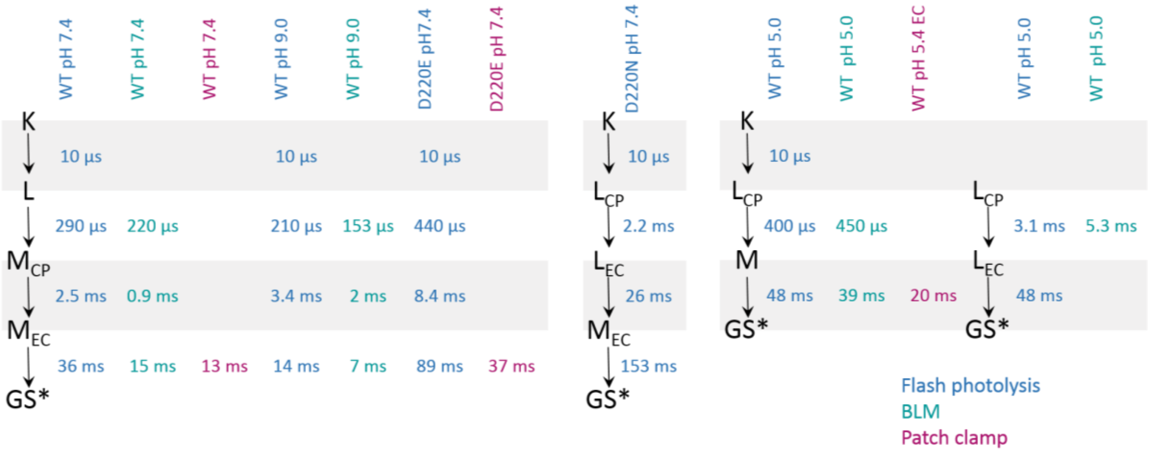

Figure S7. Summary of transition times determined by applied methods and their assignment to the photocycle intermediates.

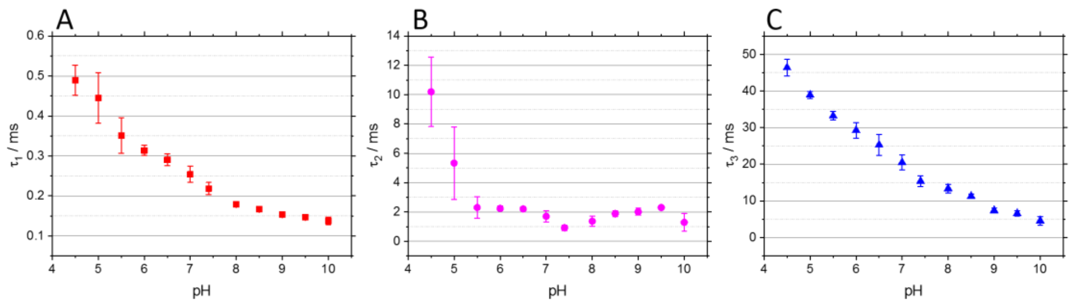

Figure S8. pH dependence of photovoltage build-up kinetics in BLM experiments. Proteoliposomes were added at pH 7.4 and the titrations were carried out by adding 1N HCl or NaOH. The results are shown as mean ± SD with n=3.

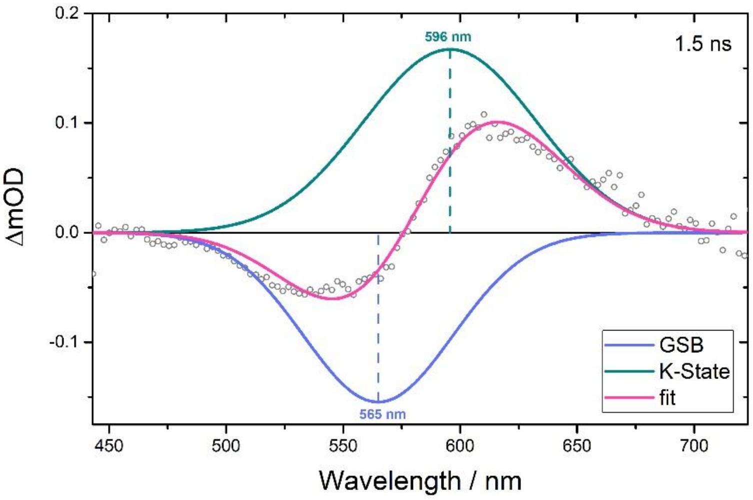

Figure S9. Weighted sum of the absorption of the K intermediate and the GSB (FWHM from absorption spectrum) through Gaussians of the transient spectrum of *Ns*XeR WT at 1.5 ns.

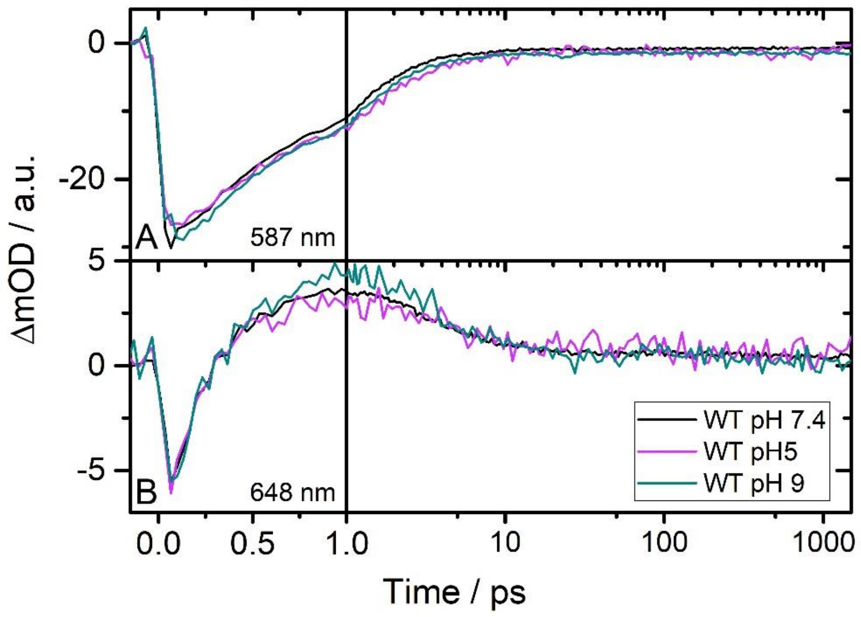

Figure S10. Transient absorption changes at probing wavelengths of 587 nm (A) and 648 nm (B) for pH 5, 7.4 and 9 of the WT.

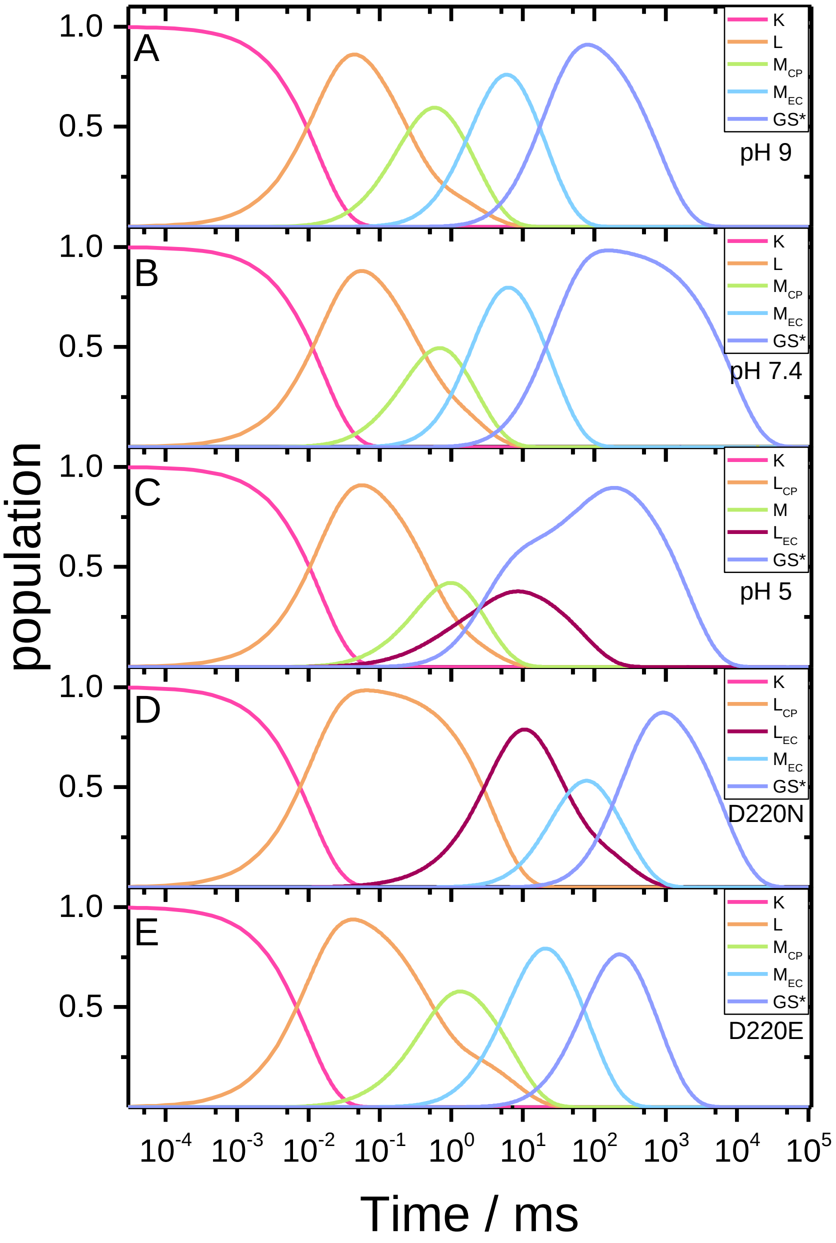

Figure S11. Populations of the various intermediates calculated from kinetic modelling.

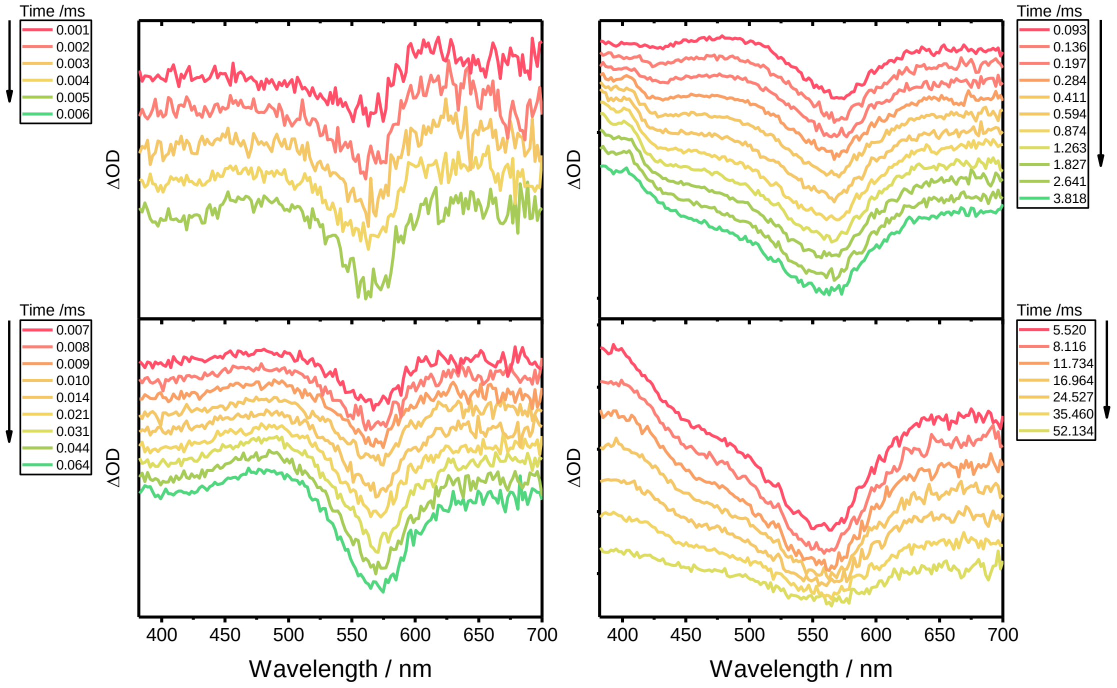

Figure S12. Difference absorption spectra of WT *Ns*XeR at indicated time delays at pH 7.4.

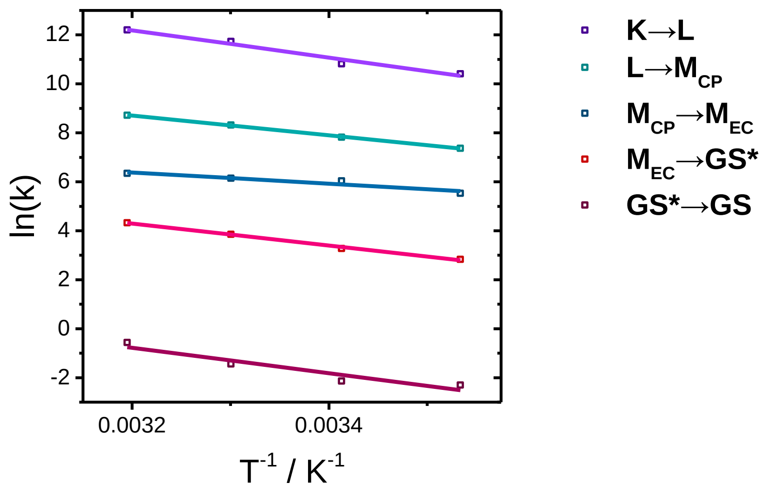

Figure S13. Arrhenius plot for WT *Ns*XeR at pH 7.4 (10-40°C).

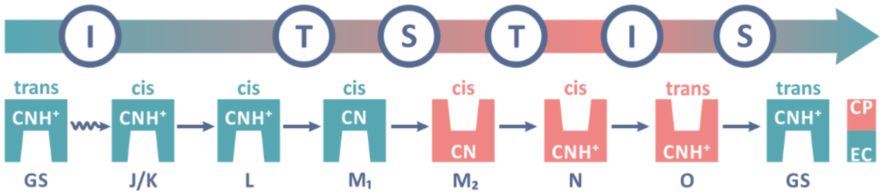

Figure S14. Sequence of the IST model for BR according to Haupts et al. [6].

Table S1. Absorption maxima and retinal Schiff base pK_a. Results are given as mean ± SD from 3 (WT, D220E) or 4 (D220N) titrations. λ_{max} of D220E is significantly different from D220N and the wild type with p < 10-3. Retinal SB pK_a values are significantly different for wild type and both mutants. p values are given in column 4 and 5.

	λ_{max} at pH 7.2 / nm	Schiff base pK_a	WT	D220N
**WT**	565.56 ± 0.18	9.56 ± 0.06	-	-
**D220N**	565.02 ± 0.35	9.84 ± 0.19	$p < 0.01$	-
**D220E**	568.13 ± 0.70	8.43 ± 0.12	$p < 10^{-9}$	$p < 10^{-11}$

Table S2. Lifetimes obtained from the GLA of the ultrafast transient data.

	WT pH5	WT pH 7.4	WT pH 9	D220N	D220E
τ_1 [ps]	0.15	0.23	0.25	0.14	0.12
τ_2 [ps]	0.61	0.63	0.51	0.63	0.81
τ_3 [ps]	3.45	3.46	2.87	3.87	4.32

Table S3. Lifetimes obtained from the GLA of the flash photolysis data.

	**WT pH5**	**WT pH 7.4**	**WT pH 9**	**D220N**	**D220E**
**τ_1 [ms]**	0.1	0.1	0.1	0.1	0.1
**τ_2 [ms]**	0.39	0.29	0.21	2.2	0.44
**τ_3 [ms]**	3.08	2.49	3.36	26.13	8.44
**τ_4 [ms]**	47.93	35.83	14.11	152.84	80.86
**τ_5[ms]**	296.32	8656.92	88.664	6582.22	812.13
**τ_6[ms]**	2067.24		808.591		

References

[1] Urmann, D., et al. Photochemical Properties of the Red-shifted Channelrhodopsin Chrimson. *Photochemistry and Photobiology* 93.3 **2017**: 782-795.

[2] Slavov, C., et al. Ultrafast coherent oscillations reveal a reactive mode in the ring-opening reaction of fulgides. *Physical Chemistry Chemical Physics* 17.21 **2015**: 14045-14053.

[3] Slavov, C., et al. Implementation and evaluation of data analysis strategies for time-resolved optical spectroscopy. *Analytical Chemistry* 87.4 **2015**: 2328-2336.

[4] Shevchenko, V., et al. Inward H+ pump xenorhodopsin: Mechanism and alternative optogenetic approach. *Science Advances* 3.9 **2017**: e1603187.

[5] Shcherbo, D., et al. Bright far-red fluorescent protein for whole-body imaging. *Nature Methods* 4.9 **2007**: 741-746.

[6] Haupts, U., et al. General concept for ion translocation by halobacterial retinal proteins: The isomerization/switch/transfer (IST) model. *Biochemistry* 36 **1997**: 2–7.
